# Optimism, Persistence, and Our Collective Crystal Ball

**DOI:** 10.4269/ajtmh.2010.10-0107

**Published:** 2010-07

**Authors:** Thomas E. Wellems

**Affiliations:** Member, American Society of Tropical Medicine and Hygiene

Thank you indeed to Christopher Plowe for the very generous introduction—I think it was perhaps too generous. Chris showed the list of fellows and students who have worked with me at National Institutes of Health (NIH), and great credit really goes to them. I have been fortunate and privileged to have been surrounded by the brilliance of all of these individuals and their accomplishments through the years. Also, Chris did not name himself in the photographs with our Malian colleagues, instead referring to himself only as one of the “less talented” fellows. Not at all! In fact, Chris was overly generous to me when he spoke of our field work in Mali on chloroquine resistance. It really was Chris' project with Abdoulaye Djimdé while I was collaborating on much of that work. So I return the credit for that to Chris, with the comment that it was particularly rewarding to see the impact on health in the villages where we collaborated with our Malian colleagues to deliver relatively inexpensive medicines that worked wonders.

Tonight marks the end of a wonderful experience for me as your *ASTMH* President. The period has left me with lasting memories, new colleagues, and friends. And it is a great satisfaction to say that this extraordinary Society continues to grow in strength and collective voice as the leading international organization of scientists and clinicians dedicated to tropical medicine and global health. *ASTMH* is alive with an abundance of positive spirit and teamwork in our membership. This year we have held our largest and most successful Annual Meeting ever, with over 3,400 registrants. We have also seen excellent progress in areas of membership, policy and advocacy, media communications, and awards program funding. Time does not allow me to thank by name everyone who made this progress possible. However, I would like to express my gratitude to some individuals in particular:

• To Sally Finney, our Executive Director, for her initiative and careful attention to our *ASTMH* activities this year. These have included the Policy and Advocacy work with Kent Campbell, the upcoming *ASTMH* Constitution and Bylaws revisions, the launch of our Public Relations Committee, the media relations contract with the MWW Group, and the coordination of our Executive Committee and Council meetings;
• To Judy DeAcetis, Lyn Maddox, Matthew Lesh, and their Sherwood Group Colleagues for their attentive care to this year's operations including the meeting arrangements, the drive to boost membership, and the wide-ranging administrative needs of our Society.
• To Chris King and the approximately 100 members of our Scientific Program Committee for their contributions of time, thought, and effort putting together a terrific Meeting Agenda this year.
• To the members of the *ASTMH* Council, especially Josh Berman, Peter Weller, Jim Kazura, Ed Ryan, Claire Panosian, Joe Vinetz, Steve Higgs, plus so many other Society members I have not named—thank you all for your generous service to *ASTMH* this year.
• Finally, to my wife Marilyn, daughter Dianne, and sons Alex and Nick and their wives Lauren and Alysen: your love and unaffected eloquence in our family have meant everything to me over the years; and thank you very much for your support through a busy and eventful 2009.

## Formative influences.

The call to give this Society's presidential address presents an opportunity to choose among almost limitless topics across subjects of tropical public health, scientific discovery, our history, and aspects of policy and advocacy tied to the goals of *ASTMH*. As an attendee of these addresses in previous years, I have enjoyed hearing previous presidents talk of their career experiences in the context of their *ASTMH* interests, particularly when these have touched on major events that have molded and restructured our perspective and activities in today's world. My thoughts in this regard returned me to some memories from my graduate student experiences at the University of Chicago. These were under two of my PhD advisors in structural biology, Robert Josephs and Paul B. Sigler. My thesis work in those years was on the fibers and crystals of deoxygenated hemoglobin that can form in erythrocytes when there is a mutation from valine to glutamate in the sixth position of the hemoglobin beta chain, namely, the sickle-cell mutation.^1^,^2^ Those experiences were among the stimuli that eventually brought me to malaria research, a personal path that I will not go into tonight. Instead, I would like to use this address to touch upon some of the profound influences of the scientist who more than any other established the field of my PhD research. He determined the structure of hemoglobin, investigated the effects of its mutations, and established core legacies of molecular medicine and molecular biology at the Cavendish Laboratory and Cambridge University in England.

## A long-term quest of fundamental discovery.

Max Perutz decided to pursue the structure of hemoglobin in 1937, at a time when genes were generally thought to be proteins, and when proteins acting as enzymes had only recently been recognized to catalyze chemical reactions in living systems. Perutz saw protein structure as a central problem of biology, and he recognized that the only way to approach it was by x-ray crystallography.^3^ He also saw hemoglobin as a molecule of immense importance and mystery, especially in the mechanisms by which it transported oxygen and carbon dioxide in the bloodstream. The existence of regular crystals of hemoglobin suggested to Perutz that the molecule's enormous number of atoms could take a specific shape and that the resulting structure would hold the key to how the molecule worked. However, in 1937, no one knew how to go about solving the structure of such a protein; indeed, there was widespread belief that the prospects for success were bleak and the goal was scarcely realistic, if not unattainable.^4^,^5^ Perutz, under generous support and advice from William Lawrence Bragg and John Desmond Bernal,^6^ nevertheless pursued the structure.

My first exposure to the optimism and persistence with which Max Perutz took on the hemoglobin problem was in my reading of a paper he contributed in 1948 in honor of the British physiologist Sir Joseph Barcroft. At that point his goal must have seemed distant indeed, for he wrote:

“On the face of it … an attempt to analyze the crystal structure of hemoglobin, or of any crystalline protein for that matter, looks about as promising as a journey to the moon.”^7^

This at a time when space travel was still a story of science fiction and the dream of reaching the moon was only the stuff of a love song. It was a marvel, I thought, to read Perutz's assessment of the hemoglobin structure problem as a possibly forlorn undertaking. He had no clear route yet to the protein structure—in fact, his model at that time would prove wrong—but he concluded in his paper that there remained “a variety of different ways of approach to the problem of protein structure. Some of these ways have proved dead ends, but many are still leading on.”^7^ His decades of single-minded pursuit of a distant goal of major importance, by paths not at all clear, not even sure to be possible, struck me as a tremendous model for a career in scientific discovery.

Perutz received critical support for his work during this period from the Medical Research Council and its Secretary Sir Edward Mellanby. In 1947, Bragg met with Mellanby and explained the difficulty of the work, the remote chances of success and, if success were achieved, the likelihood that it might well be a long time before the information would be of practical use to medicine. Mellanby accepted the risk, and he decided to support the research.^4^ After Mellanby retired in 1949, Sir Harold Himsworth became Secretary of the MRC and continued strong support for the work. Eventually, Perutz wrote a book in 1992 entitled *Protein Structure: New Approaches to Disease and Therapy*, and he dedicated it to Himsworth, writing that “[Himsworth's] foresight and courage led him to support my colleagues and my early work on protein structures when there was only the faintest hope of it ever benefitting medicine.”^8^

**Figure 1.**
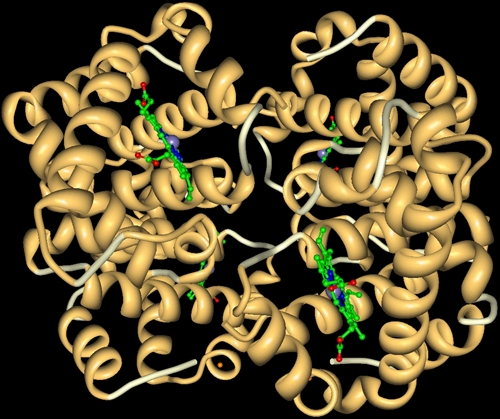
Depiction of the crystal structure of human deoxyhemoglobin at 1.74 Å resolution. The Protein Data Bank Data (ID: 2HHB) of Fermi and others^33^ were downloaded and displayed using the RCSB Protein Workshop Viewer (http://www.rcsb.org/pdb/). This figure appears in color at www.ajtmh.org.

By 1970, a year after the Apollo 11 moon landing, Perutz's determination was not only vindicated by his solution of the structure of hemoglobin (Figure 1)—he had also developed an essential description of the cooperative operations by which it functions as a breathing molecule.^9^ The exchange of gases and the physiological phenomenon of the Bohr effect were explained at the level of individual atoms, and he and Hermann Lehmann had linked clinical manifestations of a variety of genetic mutations to the positions and influence of individual amino acid substitutions.^10^

The breakthroughs of Perutz and his colleagues in crystallography not only revealed the hemoglobin molecule in all its wonder and, as he put it, its “simple beauty”^5^—the breakthroughs also set the stage for the elucidation of a world of structures from biological systems. More than 50,000 protein structures are now available over the internet from the RCSB Protein Data Bank,^11^ and applications have reached into vital areas of medicine including, for example, the development of drugs against influenza, protease inhibitors against HIV-1/AIDS, and captopril for the treatment of hypertension and heart failure.^12^

Yet Perutz's legacy extends even more broadly. As group head of the Cavendish Laboratory Unit under Medical Research Council (MRC) support, and subsequently as Chairman of the Cambridge MRC Laboratory of Molecular Biology, Perutz recruited and supported an astonishing number of brilliant colleagues.

It is still fascinating to recall the names and accomplishments from the Cavendish Unit and the Cambridge Laboratory: Watson and Crick and the DNA double helix; John Kendrew and the structure of myoglobin; Sydney Brenner and the *Caenorhabditis elegans* animal development model; Vernon Ingram and identification of the β6 hemoglobin substitution responsible for sickle-cell anemia; Hugh Huxley and the sliding filament model for muscle contraction; Cesar Milstein and the hybridoma technique for monoclonal antibody production; Aaron Klug and the structural determination of nucleic acid-protein complexes; and Frederick Sanger and sequencing methods that elucidated the first full DNA sequence of a viral genome (Phage Φ-X174).^6^,^13^

Perutz's belief in enlisting ambitious and creative scientists and giving them what they needed to succeed was an essential character of his chairmanship years. He recognized scientific creativity, he recognized what could kill it, and he understood the value of an environment in which young scientists were given the intellectual freedom to pursue their ideas—of an environment in which they were not told what to do, but they had to find out what to do by themselves.^4^,^5^

## Science and applications of science.

Perutz was deeply committed to the importance of scientific thinking and the value of basic research for a foundation of human betterment and human purposes. He used his voice as a Nobel Laureate to advocate for human rights and promote his conviction that “scientists the world over are united by a common purpose, ideally to discover Nature's secrets and put them to use for human benefit”.^14^

It was also in Perutz's nature to value research done for its own sake, in his words: to “discover the strange workings of a wonderful world.”^6^

Many of you will probably recognize the book written in 1997 by Donald Stokes, *Pasteur's Quadrant: Basic Science and Technological Innovation*.^15^ This book was also mentioned by Carlos Morel in his Commemorative Fund Lecture at last year's *ASTMH* Annual Meeting in New Orleans.^16^ Stokes' history provides an absorbing treatment of the distinctions that are frequently drawn between classifications of basic and applied research, and of how these distinctions have been incorporated into various institutional policies and government funding, particularly in the years since Vannevar Bush's 1945 report *Science—the Endless Frontier*.^17^ Stokes' thesis is that classifications of research in a one-dimensional model from basic to applied often fail to recognize the full range and motivation of investigations in science. He offers a diagram of two-dimensions in which research falls into cells or quadrants, depending upon the degree to which scientific efforts are motivated by the goals of 1) fundamental understanding and 2) considerations of use.

**Figure 2.**
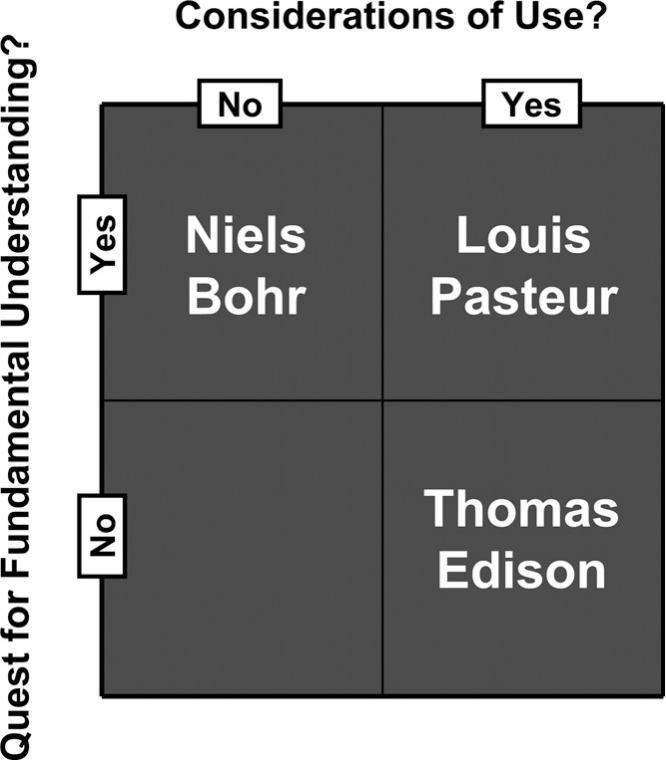
Quadrant model of research motivations proposed by Stokes.^15^

In Stokes' diagram (Figure 2), the lower right quadrant includes the work of the inventor Thomas Edison, who was famous for his quests for applications alone, without interest to develop fundamental scientific theories. The physics of Niels Bohr is placed in the upper left quadrant because of his passion for fundamental understanding on the basic level. And, in the upper right quadrant, are bodies of scientific work motivated both by curiosity about fundamental principles of nature and by the search for useful applications. Stokes named this quadrant after Louis Pasteur, who strove to understand the processes of disease at the most fundamental level, while at the same time he worked to develop ways to deal with major problems including anthrax, milk and wine spoilage, chicken cholera, rabies, and silk worm infections.

But I wonder how clearly motivations based on “considerations of use” distinguish the quadrants of Pasteur and Bohr. Pasteur once famously remarked “Il n'existe pas de sciences appliquées, mais seulement des applications de la science. [There are no such things as applied sciences, only applications of science],”^18^ also, that science and its applications are “liées entre elles comme le fruit à l'arbre qui l'a porté [linked together as fruit is to the tree that has borne it].”^19^

With these words in mind, consider Niels Bohr. He was a profound scientist, a preeminent creator of fundamental physics and a philosopher of natural phenomena, but he was likewise an orchestrator of experimental science, a builder of a renowned research Institute in Copenhagen, a helper of refugees, and a passionate advocate for international openness in the spirit of glastnost.^20^ The son of Danish physiologist Christian Bohr, for whom the Bohr hemoglobin effect studied by Max Perutz is named, Niels Bohr also looked for ways to bring physics into biology and medicine. This he did astonishingly well following a 1933 decision by Warren Weaver and the Rockefeller Foundation to focus on support for experimental biology as its primary field of interest in the natural sciences. With funding from the Rockefeller Foundation, Bohr supported his friend and colleague George de Hevesy, who pursued work with inducible radioactive tracers in Copenhagen after these were first produced by Marie Curie's daughter Irène and her husband Frédéric Joliot.^20^ Hevesy's work with artificial tracers spurred revolutionary applications in biology and founded the field of nuclear medicine.^20^,^21^

Bohr's enthusiasm for bringing physics to biology had a notable influence in another direction as well. This was an influence I first learned about in 1984, shortly after I joined NIH's malaria research group under Louis Miller, in the Laboratory of Parasitic Diseases directed by Franklin Neva. It was an exciting time: DNA sequences of the *Plasmodium falciparum* circumsporozoite protein (CSP) identified by Ruth Nussenzweig were being published,^22^,^23^ and I began a project with Russell Howard to clone and characterize the sequence of a malaria parasite gene we termed *pfhrp2*.^24^ Sequence information from the CSP gene is used today in the RTS,S recombinant malaria vaccines; and the sequences of PfHRP2 serve as the basis for a number of the rapid diagnostic tests that are now widely deployed for the detection of *P. falciparum* infection. In 1984, lambda bacteriophages, plasmids, and selected *Escherichia coli* strains were the working cloning systems in these projects. For methods and information we routinely turned to publications from the Cold Spring Harbor Laboratory, mainly *Molecular Cloning: A Laboratory Manual* published in 1982 by Tom Maniatis, Joe Sambrook, and Edward Fritsch.^25^

So, what was the influence that connects Niels Bohr, molecular biology at Cold Spring Harbor, and the cloning methods for the genes I mentioned? In a 1932 address entitled “Light and Life,” Bohr reflected on the principles of complementarity in physics and asked whether analogous principles might be needed to understand living organisms.^26^,^27^ Max Delbrück, an associate of Bohr's in theoretical physics, was so taken with the address that he decided to change the course of his career from physics to biology.^28^,^29^

Delbrück turned to biology with the view that a detailed examination of living systems might uncover paradoxical phenomena similar to those that had confronted physicists in quantum physics. He did not succeed to find such phenomena, eventually coming to the view that all dynamic systems of biology are reducible and follow the laws of physics and chemistry.^30^ But his search proved extraordinarily fruitful, as his ideas spurred fundamental investigations into the physical properties of the gene, and he and Salvador Luria opened up a new world of molecular genetics in their demonstrations of evolution by random mutation. Along with Luria, Delbrück initiated the famous phage group at the Cold Spring Harbor Laboratory (www.cshl.edu/history/Delbrück.html), in the same spirit of scientific associations that were present in the Copenhagen group around Bohr.^3^

## Past is prologue.

A half century after the molecular and genetic breakthroughs of Perutz and Delbrück, we have before us today entire genome sequences of pathogens and hosts, and we also have the understanding and wherewithal to manipulate the components of these living systems as never before. The advances we are witnessing in biology and medicine are just some of the benefits of the physical and social sciences to the human condition, as they join with advances across areas of nutrition, water and sanitation, telecommunications, agriculture, energy production, and transportation, with remarkable potential to improve health and well-being.

**Figure 3.**
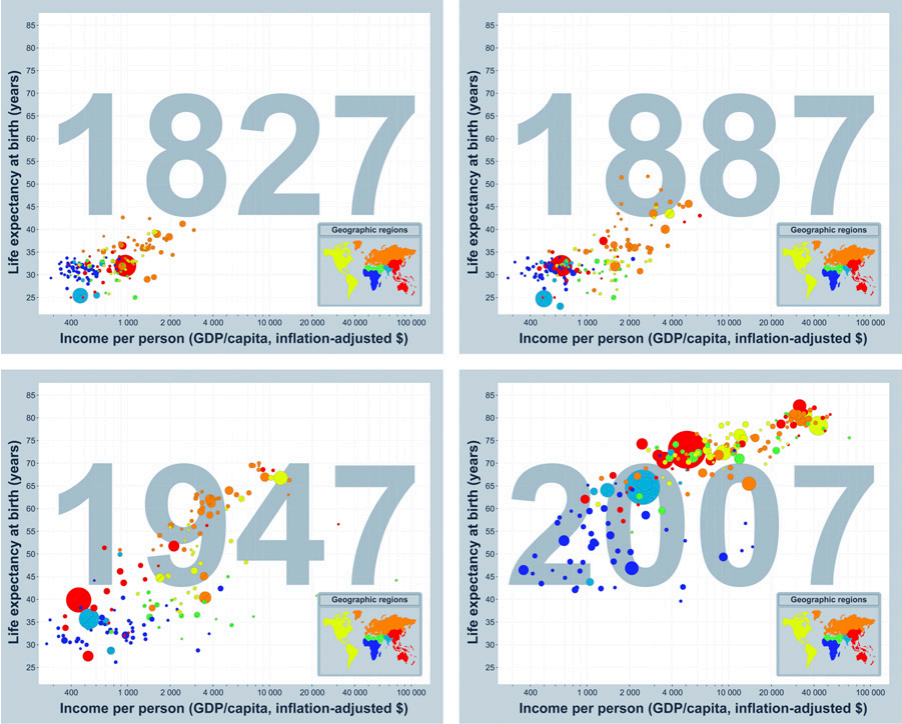
Gapminder comparisons of life expectancy at birth vs. income per person in 1827, 1887, 1947, and 2007. Colored circles represent countries from different regions of the world, and the size of each bubble represents a country's population. Plots were generated from data and software at the Gapminder web site: http://www.gapminder.org/. This figure appears in color at www.ajtmh.org.

In many countries of the world, the effects of these benefits are evident in two measures of health and well-being: life expectancy at birth and income per person. Some of you are probably familiar with the trends analysis of these measures by Hans Rosling, Professor of International Health at the Karolinska Institutet and Director of the Gapminder Organization. His presentations bring global health and economic trends to life. Figure 3 shows a Gapminder series comparing life expectancy at birth and income per person from 1827, a year roughly between the time Edward Jenner began using the cowpox vaccine against smallpox and the time John Snow removed the Broad Street pump handle in his great epidemiological advance against cholera. From a life expectancy range of 25 to 40 years in the early 1800s, this expectancy has increased for most countries, increasing first in the industrialized countries, for example in Europe and North America. And life expectancy then shows great improvements in some other large populations, for example in India and China, where about 50 years ago life expectancy began increasing at a rate almost twice as fast as the earlier rates in Europe and North America. For India and China the increases in life expectancy began some years before the increases in prosperity. As Rosling points out for these statistics, “you get wealthy faster if you're healthy first.”

There is momentum in these gains, and it is in the right direction. We know that in many countries of Africa and some other regions of the world, overall improvements have lagged. But past is prologue, and the momentum of the gains tells us we can be confident that better conditions of health will be followed by increases in prosperity.

I am proud to say it is on these challenges of health that our *ASTMH* membership focuses:

• We work as a worldwide organization to prevent and control infectious and other diseases that disproportionately afflict the global poor.
• Our goals include advancing research on tropical diseases, fostering international scientific collaborations, promoting science-based policy, and supporting education and career development of professionals in tropical medicine and global health.

President Obama this year pointed out that “Science is more essential for our prosperity, our security, our health, our environment, and our quality of life than it has ever been before” and “… many of the challenges that science and technology will help us meet are global in character.”^31^ Science is at the heart of our goals in *ASTMH*, and the innovations that follow from investments in science, from fundamental discoveries and their applications, will give us vital means to meet these goals.

A report recently announced by the National Research Council's Board on Life Sciences, “*A New Biology for the 21st Century*,”^32^ opens by asking that we imagine a world where:

• there is abundant, healthful food for everyone
• the environment is resilient and flourishing
• there is sustainable, clean energy
• good health is the norm

In calling for a national initiative to address these goals, the report reaffirms the role of fundamental research endeavors and recommends that they be met by integrated, interdisciplinary efforts across the biological and physical sciences. These recommendations echo the approaches of Perutz, Bohr, and their colleagues as they brought concepts of physical sciences and new computational technology to great advances in biology a half century ago. Scientific discovery and its applications give us the platform for continued progress that will improve our collective ability to control disease, improve economic production, and nourish and support our world's populations. Even in the face of issues of climate change, global population burden, environmental depredations, and emerging disease threats, science and its processes give us capacities scarcely imaginable just a few decades ago; and future capacities will come that we can scarcely imagine today.

The ways of thinking that have enabled us to travel to the moon, and have given us new ways to understand ourselves and the universe, have the power to meet these goals. But we must stay energetic in our endeavors to discover and learn. Max Perutz once said of his optimism and persistence in the face of seemingly intractable difficulties toward his goal: “As always, I was driven on by unrealistic expectations.”^3^ He harnessed new knowledge, information, and technological capacities to meet those expectations. If there seem to be any advances in global health or in prosperity that seem likewise unrealistic, I would counter that momentum of the gains is with us, and new discoveries and their applications continue to give us tremendous potential.

Our endeavors toward these discoveries and applications will forever remain worthy of our most ambitious dreams.
